# Influence of the COVID-19 pandemic on the timing of surgical triage, tumor stage, and therapy of patients with colon carcinoma

**DOI:** 10.1007/s00384-023-04430-9

**Published:** 2023-05-31

**Authors:** Fiona Speichinger, Ann-Kathrin Berg, Ani Stoyanova, Ioannis Pozios, Florian Loch, Johannes C. Lauscher, Katharina Beyer, Nadia Slavova, Christian Schineis

**Affiliations:** Department of General and Visceral Surgery, Charité Universitätsmedizin, Berlin, Germany

**Keywords:** COVID-19 pandemic, SARS-CoV-2, Colon carcinoma, Endoscopy, Surgical triage

## Abstract

**Purpose:**

With the onset of the COVID pandemic in Germany in March 2020, far-reaching restrictions were imposed that limited medical access for patients. Screening examinations such as colonoscopies were greatly reduced in number. As rapid surgical triage after diagnosis is prognostic, our hypothesis was that pandemic-related delays would increase the proportion of advanced colon cancers with an overall sicker patient population.

**Methods:**

A total of 204 patients with initial diagnosis of colon cancer were analyzed in this retrospective single-center study between 03/01/2018 and 03/01/2022. Control group (111 patients, pre-COVID-19) and the study group (93 patients, during COVID-19) were compared in terms of tumor stages, surgical therapy, complications, and delays in the clinical setting. The data were presented either as absolute numbers or as median for constant data.

**Results:**

A trend towards more advanced tumor stages (T4a *p* = 0.067) and a significant increase of emergency surgeries (*p* = 0.016) with higher rates of ileus and perforation (*p* = 0.004) as well as discontinuity resections (*p* = 0.049) during the pandemic could be observed. Delays in surgical triage after endoscopic diagnosis were seen during the 2nd lockdown (02/11/20–26/12/20; *p* = 0.031).

**Conclusion:**

In summary, the results suggest delayed treatment during the COVID-19 pandemic, with the infection pattern of COVID appearing to have a major impact on the time between endoscopic diagnosis and surgical triage/surgery. Adequate care of colon cancer patients is possible even during a pandemic, but it is important to focus on structured screening and tight diagnosis to treatment schedules in order to prevent secondary pandemic victims.

## Introduction

In the beginning of 2020, the World Health Organization (WHO) declared a pandemic due to the rapid worldwide spread of a new virus: SARS-CoV-2 (severe acute respiratory syndrome coronavirus-2) [1, 2]. In Germany, the pandemic led to far-reaching restrictions, including several complete lockdown periods between March 2020 and May 2021. It has already been shown that there were restrictions on access to medical care during the pandemic. The number of referrals with subsequent timely physician consultations and surgeries decreased [3, 4]. Screenings for a wide variety of cancers declined during the pandemic [5, 6]. This is of enormous importance, especially for colon carcinoma, since screening without clinical symptoms is indispensable. This is further supported by the fact that the incidence of colon cancer in Germany has decreased significantly since the introduction of structured screening [7]. Quality-assured colonoscopy has the highest specificity and sensitivity [8, 9] for detecting early precursor lesions (i.e., adenomas) and is therefore indispensable, even in times of pandemics. Likewise, rapid surgical presentation after confirmation of the diagnosis is decisive for therapy and prognosis. Of concern in this context are the decreasing numbers of colonoscopies and fecal occult blood testing (FOBT) [10, 11], resulting in a delay in the treatment of colorectal cancer [12, 13]. Since during the COVID pandemic the health care system repeatedly reached the limits of its capacity with each wave of the pandemic, and lockdowns as well as individual fear of infection resulted in fewer physician visits, the primary hypothesis of our study is that an accumulation of more advanced tumor stages resulted due to an increase in the time to diagnosis and time from diagnosis to surgical therapy during the pandemic.

## Methods

A total of 204 patients at the Department of General and Visceral Surgery at the Charité University Hospital (Campus Benjamin Franklin, Berlin, Germany) with an initial diagnosis of colon cancer between 01/03/2018 and 28/02/2022 were retrospectively enrolled in the study. Patients for second opinion, appendix, and rectum carcinoma as well as patients younger than 18 years of age were excluded from the analysis. Patients diagnosed and treated at our hospital during the COVID pandemic (01/03/2020–28/02/2022) were assigned to the study group, and patients diagnosed and treated before the pandemic during the inclusion period (01/03/2018–29/02/2020) were assigned to the study group. A total of 111 patients were included in the pre-COVID-19 pandemic group (control group) and 93 patients were included in the COVID- 19 pandemic group (study group). A total of 178 patients underwent surgery—conventional open or laparoscopic resection (93 patients in the control group; 85 patients in the study group). All patients of the study group had been tested for SARS-CoV-2 and none of them had tested positive for coronavirus, neither at the time of diagnosis nor at the time of surgery. Clinicopathological characteristics such as age, sex, and pre-existing disease as well as tumor localization were determined for each patient and are summarized in Table 1. To detect differences in treatment delays, the starting point for both groups was an endoscopically confirmed colon carcinoma. The study design has been reviewed and approved by the Ethics Committee, Charité Universitätsmedizin Berlin (number of proposal EA4/007/23). All study participants gave their written consent to the study.

**Table 1:** Clinicopathologics of all patients

	**Control group**	**COVID-19 pandemic**		***p*-value**
	(*n* = 111)	(*n* = 93)		
Age	74.0 (22.1–28.3)	72.0 (57.5–82.5)		0.919
BMI	25.0 (64.0–79.0)	24.8 (22.6–27.6)		0.737
Female sex	35 (31.5%)	33 (35.5%)		0.552
Endoscopic diagnosis	101 (91%)	73 (78.5%)		0.012*
**Comorbidities**				
Diabetes mellitus	23 (20.7%)	12 (12.9%)		0.141
Cardiovascular risk factors	72 (64.9%)	60 (64.5%)		0.959
COPD, asthma	9 (8.1%)	8 (8.6%)		0.899
Smoking	18 (16.2%)	27 (29.0%)		0.316
Renal failure	12 (10.8%)	8 (8.6%)		0.584
Chronic inflammatory bowel disease	6 (5.4%)	8 (8.6%)		0.282
**Tumor localization**				0.857
Cecum/ascending colon	44 (39.6%)	38 (40.9%)		0.860
Right flexure	2 (1.8%)	5 (5.4%)		0.163
Transverse colon	18 (16.2%)	8 (8.6%)		0.105
Descending colon	6 (5.4%)	9 (9.7%)		0.245
Sigmoid colon	41 (36.9%)	33 (35.5%)		0.830

Age and BMI are shown as median with interquartile range in brackets. Remaining data is shown as absolute number with percentages in brackets

*BMI* body mass index, *COPD* chronic obstructive pulmonary disease

*p-values < 0.05 were considered significant

SPSS 27 (IBM, Armonk, NY) was used for the statistical analysis. Constant data did not show Gaussian distribution and therefore presented as median with lower/upper quartiles. Median gender distribution and age were calculated. Significance levels were calculated using non-parametric tests for non-dependent samples. *p*-values < 0.05 were considered significant.

## Results

### Demographic data

Clinicopathological data are summarized in Table 1. In total, 111 patients with initial diagnosis of colon cancer were included in the control group. The median age was 74 years. Thirty-five (31.5%) of them were female. Ninety-three patients with colon cancer were included in the study group with a median age of 72 years. Thirty-three (35.5%) of them were female. No significant differences in comorbidities and tumor localization were found between the groups. Significantly more patients in the control group received their diagnosis endoscopically (*p* = 0.012).

Ninety-three patients of the control as well as 85 patients of the study group underwent surgery (Table 2). Laparoscopic surgery was performed in 66.7% (control group) respectively 55.3% (study group). Conversion to open surgery was performed in 14% of the patients in the control group and 12% in the study group. No statistical difference between the groups could be found (*p* = 0.661). Significantly more patients received a stoma (*p* = 0.016) in the study group with a higher proportion of terminal stomata (*p* = 0.049). In contrast, there was no difference in creation of protective stomas (*p* = 0.293). Furthermore, no significant differences between both groups regarding postoperative complications (Clavien-Dindo [14]) and preoperative morbidity (classified according to the American Society of Anesthesiologists (ASA)) [15] could be found. Overall, more emergency surgeries were performed in the study group with significantly fewer elective surgeries compared to the control group (*p* = 0.016). Moreover, significantly more patients with tumor-related ileus and perforation were treated in the study group (*p* = 0.004). Consequently, significantly more patients in the study group had to be admitted to the intensive care unit postoperatively (*p* = 0.030). In contrast, there was no difference in the overall length of hospital stay (LOS).

**Table 2:** Characteristics of the operated patients

	**Control group** (*n* = 93)	**COVID-19 pandemic** (*n* = 85)	*p*-value
Laparoscopy	62 (66.7%)	47 (55.3%)	0.121
Open surgery	31 (33.3%)	38 (44.7%)	
Conversion	13 (14.0%)	10 (12.0%)	0.661
Prevalence of stoma	29 (31.2%)	42 (49.4%)	0.016*
Terminal stoma	14 (15.1%)	23 (27.1%)	0.049*
Protective stoma	15 (16.1%)	19 (34.1%)	0.293
Ileus/perforation	4 (4.3%)	15 (17.6%)	0.004*
Postoperative ICU	26 (28.0%)	37 (43.5%)	0.030*
Days ICU	0 (0.0–1.0)	0 (0.0–3.0)	0.190
Length of hospital stay (LOS)	8 (6.5–18.5)	9 (6.0–14.5)	0.190
**ASA classification**			
ASA 1	8 (8.6%)	7 (8.2%)	0.930
ASA 2	45 (48.4%)	37 (43.5%)	0.517
ASA 3	38 (40.9%)	33 (38.8%)	0.782
ASA 4	2 (2.2%)	7 (8.2%)	0.065
ASA 5	0	1 (1.2%)	0.296
**N classification**			
N0	0	0	
N1	2 (2.2%)	5 (5.9%)	0.202
N2	4 (4.3%)	10 (11.8%)	0.091
N3	2 (2.2%)	4 (4.7%)	0.347
N4	3 (3.2%)	3 (3.5%)	0.775
N5	82 (88.2%)	63 (74.1%)	0.016*
**Clavien-Dindo**			
I	5 (5.4%)	8 (9.4%)	0.282
II	5 (5.4%)	1 (1.2%)	0.129
III	11 (11.8%)	6 (7.1%)	0.304
IV	10 (10.8%)	14 (16.5%)	0.239
V	5 (5.4%)	5 (5.9%)	0.853

N classification: surgery urgency (N0: immediately, N1 < 1 h, N2 < 6 h, N3 < 12 h, N4 < 24 h, N5 elective); Clavien-Dindo (I: deviation of the normal, II: pharmacological treatment, III: surgical/interventional therapy, IV: life threatening therapy, V: death). Days in ICU as well as LOS are shown as median with interquartile range in brackets. Remaining data is shown as absolute number with percentages in brackets

*ICU* intensive care unit, *ASA* Society of Anesthesiologists

*p-values < 0.05 were considered significant

### Timeline between diagnosis and therapy

Table 3 displays the timeline as a median of days between endoscopic diagnosis, first surgical triage, and following surgery in relation to the pandemic situation in Berlin. Significantly more time elapsed between endoscopic diagnosis and surgical triage during the second lockdown compared to the pre-pandemic period (*p* = 0.031). Similarly, a trend toward delay during the second lockdown was evident compared to the pandemic without lockdown (*p* = 0.057). No further significant differences were found between the study group during the three lockdowns and the control group. Likewise, days between endoscopic diagnosis and surgery showed no significant differences (*p* = 0.508).

**Table 3:** Timeline diagnosis-therapy

	**Control**	**COVID-19 pandemic**	***p*-value**
	(*n* = 101)	(*n* = 73)	
**Days endoscopic diagnosis—surgical triage**			
Complete time period	4 (1.0–10.0)	6 (2.0–12.5)	0.277
No lockdown		5.5 (1.25–10.0)	vs. 2.LD:0.057
1.German lockdown 22/03/20–04/05/20		7 (1.5–12.5)	
2.German lockdown 02/11/20–26/12/20		16 (4.0–18.0)	vs. C: 0.031*
3.German lockdown 27/12/20–09/05/21		5 (1.75–12.75)	
	(*n* = 88)	(*n* = 68)	
**Days endoscopic diagnosis—surgery**			
Complete time period	12 (7.0–21.0)	14 (6.0–27.0)	0.508
No lockdown		12.0 (5.0–27.0)	
1.German lockdown 22/03/20–04/05/20		18.0 (5.0–21.25)	
2.German lockdown 02/11/20–26/12/20		21.0 (6.0–31.75)	
3.German lockdown 27/12/20–09/05/21		19.0 (4.0–30.0)	

Data is shown as median with interquartile range in brackets. Lockdown date (dd/mm/yy). *p-values < 0.05 were considered significant. No significant difference was shown between the different lockdown groups. For detailed significance values, see Table 5 in the Appendix

### Tumor characteristics

Table 4 shows the different tumor stages of both groups (according to the 8th American Joint Committee on Cancer (AJCC)/Union Internationale Contre le Cancer (UICC) Tumor-Node-Metastasis (TNM) staging system) [16–18]. Considering the tumor stage, no significant difference between both groups could be found. But regarding T4 stages, a noticeable trend (*p* = 0.067) towards more T4 stages in the study group is evident. No significant differences regarding the nodal stages could be seen. Considering the UICC stages, a noticeable trend towards early stages in the control group could be seen (*p* = 0.063).

**Table 4:** TNM/UICC stages

	**Control**	**COVID-10 pandemic**	***p*-value**
	(*n* = 111)	(*n* = 93)	
**T stage**	*n *= 91	*n *= 82	
T1	13 (14.3%)	9 (11.0%)	0.549
T2	20 (22.0%)	12 (14.6%)	0.241
T3	49 (53.8%)	46 (56.1%)	0.662
T4a	5 (5.5%)	11 (13.4%)	**0.067**
T4b	4 (4.4%)	4 (4.9%)	0.857
**N stage**	*n *= 92	*n *= 83	
N0	55 (65.5%)	47 (56.6%)	0.737
N1a	7 (8.3%)	10 (12.0%)	0.312
N1b	12 (14.3%)	12 (14.5%)	0.765
N1c	4 (4.8%)	2 (2.4%)	0.491
N2a	6 (7.1%)	5 (6.0%)	0.907
N2b	8 (9.5%)	7 (8.4%)	0.968
**UICC stage**	*n *= 104	*n *= 90	
UICC 0	2 (1.9%)	1 (1.1%)	0.648
UICC I	26 (25.0%)	12 (13.3%)	**0.063**
UICC II	27 (26.0%)	32 (35.6%)	0.218
UICC III	31 (29.8%)	29 (32.2%)	0.756
UICC IV	18 (17.3%)	16 (17.8%)	0.96

Data is shown as absolute number with percentages in brackets. *p*-values < 0.05 were considered significant

## Discussion

Early detection of colorectal cancer determines the prognosis of the disease. Therefore, screening colonoscopies and, if necessary, prompt surgical presentations are essential. Due to decreasing numbers of colonoscopies and reduced physician visits, caused by restrictions in the health care system and in social restrictions during the COVID-19 pandemic [3, 10, 11], the number of more advanced tumor stages should have increased.

In addition, the COVID pandemic has had a general impact on the number of tumor operations performed. For example, a decrease of up to 40% in rectal cancer surgeries has been observed. It seems that mainly incisional cancers are affected; this is especially true for colorectal cancer [19–22].

This study is designed to assess the impact of the pandemic and lockdowns on the timing of diagnosis and treatment of colon cancer and to analyze changes in the patient population with respect to disease severity and clinical presentation. To our knowledge, this study is the only study at this time to examine the impact of the pandemic, lockdowns, viral variants, and vaccinations over the entire course of the pandemic and in such a large patient population. Indeed, we saw a higher percentage of T4a tumors in the study group, as well as a higher percentage of UICC I stages in the control group. Significant differences were not seen, but a clear trend was recognizable. Raduiovic et al. [23] also saw an increased proportion of T4b tumors and UICC IIC in the pandemic group with an increased proportion of UICC IIA in the control group. Like the Serbian group, we did not see any difference regarding nodal status. The current literature is contradictory here. A Brazilian group detected significant less nodal negative patients in the study group, but higher numbers of T4 tumors without significance. Informative value is low since they analyzed only clinical TNM stages [24]. Still, others saw no difference at all in the TNM classification [25] but analyzed only elective surgery of colorectal cancer. A Korean group around Choi et al. [26] saw no significant differences in TNM stages between the control and pandemic groups. However, there were more adherent adjacent organs with significantly more extensive surgery during the pandemic compared to the control time before, as well as a significantly higher number of lymphatic vessel invasions. All in all, this speaks for a more advanced tumor disease during the pandemic period. It should be noted that all the studies discussed here included colon as well as rectum carcinoma. We consider this to not be a feasible approach in study design since the treatment of rectal cancer follows a completely different time sequence and virus variants, lockdowns, and ICU occupancies during the pandemic could bias the data. Moreover, the observation period in all the studies mentioned above is considerably shorter since those studies had been published before the pandemic was declared over.

Furthermore, we assumed an increase in sicker patients with more postoperative complications. The study group showed a significantly increased rate of stomata in general, and significantly more terminal colostomies in particular. This can be explained by a significantly increased rate of discontinuity resections due to a higher number of emergencies. Significantly more patients had to be operated due to tumor ileus or tumor perforation in our cohort. This also resulted in a higher number of patients requiring postoperative intensive care. Nevertheless, both groups did not differ in terms of overall hospital stay, clinicopathological profile, or ASA. The number of postoperative complications did not increase during the pandemic. In contrast to the present study, the Serbian group did not see any difference to the ostomy appliance [23]. Differences for this are conceivable due to the significantly lower number of included patients in the study group (49 vs. 85) and an unclear proportion of emergency surgeries within those patients. Regarding the surgical procedure (open versus laparoscopic), we saw no differences as well as conversion rates were also not increased under pandemic conditions in our collective. The current literature is divided on this point. Choi et al. [26] saw lower rates of laparoscopic resections compared to the time before the pandemic due to more extensive surgeries with higher rates of affected neighboring organs. In contrast, like our findings, Raduiovic et al. did not see increased rates of open resections [23]. In both studies, as already mentioned, the proportion of emergencies is unclear. In addition, the proportion of laparoscopic resections for colorectal cancer varies widely worldwide. Therefore, it is possible that at hospitals that generally operate less laparoscopically, the step to conversion or the decision to perform primarily open surgery is taken rather quickly [27]. In contrast, the probability of open surgery, even for locally advanced carcinomas, is significantly lower in hospitals with a high level of laparoscopic expertise [28, 29]. Our clinic has a high level of expertise in laparoscopic colon surgery, which is why even locally advanced carcinomas are primarily approached laparoscopically if at all possible.

**Fig. 1:**
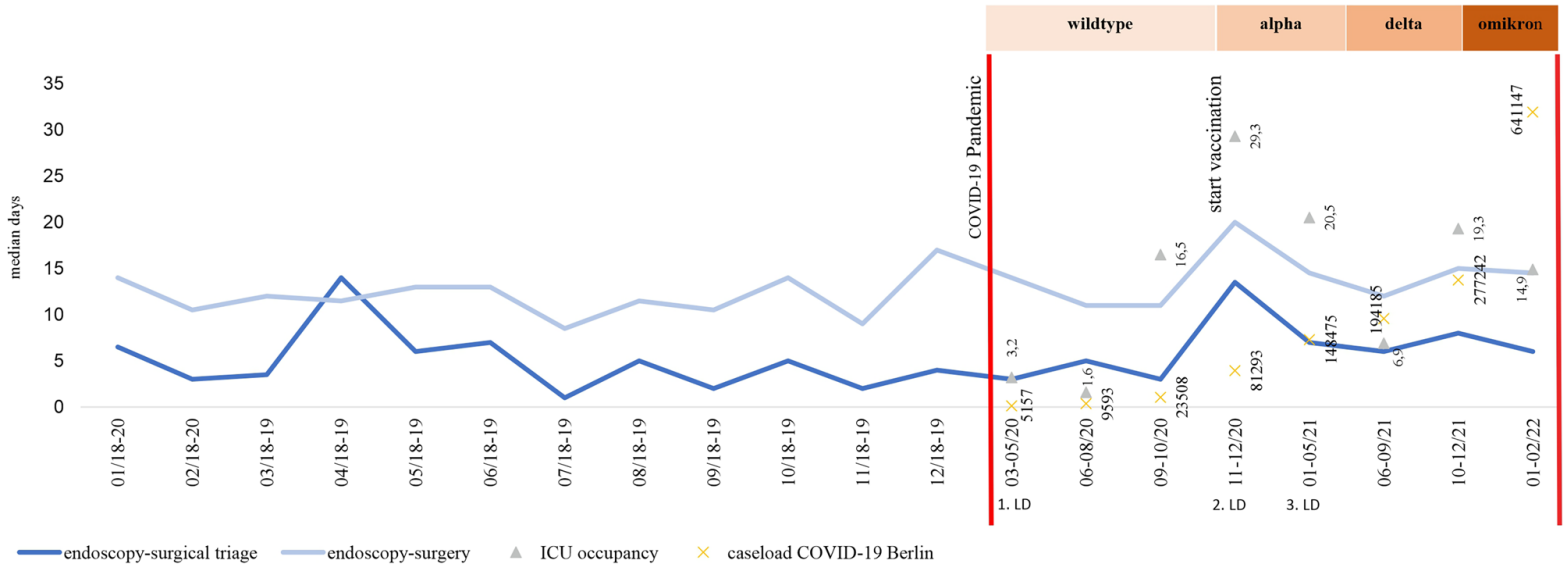
Timeline diagnosis-therapy. Timeline as median days between endoscopic diagnosis and surgery as well as endoscopic diagnosis and first surgical triage. Red bar indicates begin and end of the COVID-19 pandemic. Black bars indicate lockdowns 1–3. Start of vaccination was 12/2020. Triangle shows caseload in Berlin. Variant of coronavirus over time (RKI, Statista 2022) [30]. Data on intensive care capacity and case load in Berlin during the COVID-19 pandemic according to LAGeSo (Landesamt für Gesundheit und Soziales Berlin) [31]

Since a delay in the course of treatment of colorectal cancer during the COVID-19 pandemic could be detected [3, 13], we analyzed our patient population with respect to delayed surgical treatment by determining the time between endoscopic diagnosis, surgical triage, and surgery. Since we only saw a significant delay during the 2nd lockdown (Table 3), we took a closer look at the pandemic conditions such as vaccination, COVID variants, and ICU occupancy over time (Fig. 1). With the onset of the pandemic in March 2020, there was an overall increase in median days between endoscopic diagnosis and initial surgical triage as well as endoscopic diagnosis and surgery (Table 3). Changes during the pandemic due to changes in viral variants with increasing numbers of cases [30], available vaccine, and ICU occupancy [31] also affected the time between diagnosis and therapy. The longest delays between diagnosis and therapy could be attributed to the change of virus variants. According to our data, it appears that the infection pattern of the COVID-19 pandemic has more influence on the delay of the diagnostic and treatment than imposed lockdowns. As ICU occupancy increases, the time between endoscopic diagnosis and surgical triage appears to lengthen in our data. Nevertheless, we could not see compelling effects on time from surgical triage to surgery. An explanation could be the overload with other patients in other departments of our clinic. Also conceivable could be a delayed communication on the part of the gastroenterology respectively the internal medicine due to strongly increasing numbers of internal patients and altogether high staff shortage rates due to infections with COVID-19 themselves, especially during alteration of virus variants with changes in the pandemic event. The extended time between endoscopic diagnosis, surgical triage, and surgery, especially at the end of 2021 and beginning 2022, can also be attributed to postponed elective surgery for other diseases, which caused an increased workload.

Limitations of the present study include the nature of retrospective collected data as well as the lack of analysis of actual colonoscopies performed in and especially outside our institution during the pandemic. Since the Charité was the backbone of the corona care in Berlin and the surrounding states, it is possible that more colon carcinomas were operated on at smaller hospitals, which had transferred the care of corona patients to a large extend to the Charité. This could have led to a shift of more severe cases at our institution but is rather unlikely, since the distribution of intensive care beds was organized centrally and all patients requiring intensive care were distributed among all available beds in the Berlin area and neighboring states. Still, not all patients require intensive care postoperatively. This is especially true for patients with low tumor stages and few comorbidities that possibly could be operated on in a higher frequency in smaller institutions. Nevertheless, staff shortages and bed reductions in peripheral wards affected all hospitals. Therefore, we do not think that our data is biased in respect to the severity of disease and that the data considering the shift toward more advanced stages and more emergency resections are valid. Still, since we do not know how many colonoscopies were performed as outpatient and inpatient procedures during the pandemic, we are not able to make a direct statement about the impact of the pandemic on colon carcinoma screening in general.

Establishment of action strategies for future pandemics is in demand. For example, it has been shown that broad, standardized COVID-19 screening ensures timely patient care while minimizing risk to staff and preventing infection of patients within the hospital [32]. Continuous self-assessment by hospitals using scoring systems could also contribute to better preparation for future pandemics [33].

## Conclusion

The COVID pandemic had a significant influence on the diagnostic and therapeutic process of colon cancer. We saw a trend toward more advanced tumor stages and a significant increase of emergency surgeries with higher rates of discontinuity resections during the pandemic. A delay in surgical triage after endoscopic tumor detection was exclusively seen in the 2nd lockdown. It appears that the infection pattern of the COVID-19 pandemic has more influence than imposed lockdowns. In summary, adequate care of patients with colon cancer was possible in our hospital even under pandemic conditions but in similar situations in the future, a special focus must be imposed on tumor screenings, and tight diagnostic-to-treatment schedules have to be followed in order for those patients not to become secondary pandemic victims.0.004* 0.003*

**Table 5:** Overview and comparison between the lockdown phases

	Total numbers					*p*-values									
	1. LD	2. LD	3. LD	no LD Covid	Pre Covid	1. vs. 2. LD	1. vs. 3. LD	1. vs. no LD Covid	1. LD vs. pre Covid	2. vs. 3. LD	2. vs. no LD Covid	2. vs. pre Covid	3. vs. no LD Covid	3. vs. pre Covid	no LD Covid vs. pre Covid
Laparoscopy	2	3	5	37	62	0.575	0.529	0.234	0.100	1.0	0.681	0.407	0.606	0.296	0.314
Open surgery	4	3	5	26	31	0.575	0.529	0.234	0.100	1.0	0.681	0.407	0.606	0.296	0.314
Conversion	0	1	1	8	13	0.317	0.439	0.357	0.328	0.705	0.784	0.855	0.811	0.728	0.819
Prev. stoma	2	2	5	33	29	1.0	0.529	0.376	0.927	0.529	0.376	0.927	0.889	0.241	0.009*
Term. stoma	2	1	2	18	14	0.523	0.564	0.807	0.241	0.873	0.536	0.915	0.575	0.683	0.041*
Prot. stoma	0	1	3	15	15	0.317	0.150	0.180	0.288	0.873	0.694	0.972	0.675	0.275	0.234
Ileus/perforation	1	0	3	11	4	0.280	0.756	0.988	0.125	0.179	0.249	0.611	0.566	0.004*	0.003*
ASA classification															
ASA 1	0	0	3	4	8	1.0	0.150	0.528	0.456	0.150	0.528	0.456	0.019*	0.038*	0.606
ASA 2	3	2	1	31	45	0.575	0.083	0.971	0.939	0.262	0.460	0.476	0.021*	0.021*	0.920
ASA 3	2	4	5	22	38	0.269	0.529	0.938	0.717	0.529	0.128	0.217	0.362	0.579	0.456
ASA 4	1	0	1	5	2	0.317	0.705	0.472	0.045*	0.439	0.477	0.718	0.826	0.163	**0.088**
ASA 5	0	0	0	1	0	1.0	1.0	0.758	1.0	1.0	0.758	1.0	0.690	1.0	0.224
N classification															
N1	1	0	0	4	2	0.317	0.197	0.355	0.045*	1.0	0.528	0.718	0.416	0.641	0.182
N2	0	0	2	8	4	1.0	0.257	0.444	0.728	0.257	0.444	0.728	0.485	0.049*	0.095
N3	0	0	1	3	2	1.0	0.439	0.587	0.718	0.439	0.587	0.718	0.502	0.163	0.365
N4	0	0	0	3	3	1.0	1.0	0.752	0.657	1.0	0.752	0.657	0.684	0.566	1.0
N5	5	6	7	45	82	0.317	0.564	0.536	0.726	0.150	0.130	0.374	0.927	0.113	0.009*
Clavien															
Classification															
I	0	0	2	6	5	1.0	0.257	0.424	0.562	0.257	0.424	0.562	0.350	0.082	0.295
II	0	0	0	1	5	1.0	1.0	0.754	0.562	1.0	0.754	0.562	0.686	0.454	0.234
III	0	0	1	5	11	1.0	0.439	0.469	0.374	0.439	0.469	0.374	0.850	0.865	0.472
IV	1	3	1	9	10	0.241	0.705	0.901	0.657	**0.083**	0.033*	0.006*	0.691	0.942	0.462
V	1	0	1	3	5	0.317	0.705	0.250	0.264	0.439	0.581	0.562	0.521	0.555	0.901
T stage															
T1	0	1	1	7	13	0.317	0.439	0.384	0.331	0.705	0.710	0.847	0.892	0.737	0.670
T2	1	2	2	7	20	0.523	0.873	0.710	0.789	0.564	0.137	0.492	0.456	0.925	0.117
T3	4	2	4	36	49	0.269	0.317	0.717	0.491	0.796	0.229	0.374	0.264	0.468	0.401
T4a	1	0	3	7	5	0.317	0.564	0.710	0.259	0.150	0.384	0.564	0.121	0.006*	0.163
T4b	0	0	0	4	4	1.0	1.0	0.521	0.608	1.0	0.521	0.608	0.408	0.508	0.528
N stage															
N0	3	5	5	34	55	0.241	1.0	0.789	0.661	0.197	0.194	0.242	0.737	0.579	0.677
N1a	1	0	0	9	7	0.317	0.197	0.901	0.428	1.0	0.316	0.488	0.197	0.371	0.152
N1b	1	1	2	8	12	1.0	0.873	0.809	0.792	0.873	0.809	0.792	0.5565	0.536	0.970
N1c	0	0	0	2	4	1.0	1.0	0.655	0.606	1.0	0.655	0.606	0.564	0.506	0.749
N2a	1	0	2	2	6	0.317	0.873	0.133	0.347	0.257	0.655	0.523	0.035	0.130	0.387
N2b	0	0	1	6	8	1.0	0.439	0.424	0.456	0.439	0.424	0.456	0.987	0.882	0.795
UICC stage															
UICC0	0	1	0	0	2	0.355	1.0	1.0	0.733	0.733	0.002*	**0.052**	1.0	0.616	0.266
UICCI	0	3	2	7	26	0.079	0.323	0.365	0.163	0.187	0.034*	0.300	0.763	0.446	0.046*
UICCII	3	1	3	25	27	0.181	0.253	0.531	0.201	0.648	0.235	0.493	0.341	0.823	0.132
UICCIII	2	1	4	22	31	0.435	0.913	0.980	0.855	0.429	0.295	0.382	0.831	0.943	0.583
UICCIV	1	1	4	10	18	0.909	0.528	0.934	0.968	0.429	0.939	0.838	0.190	0.244	0.744

	Median (IQR)					*p-*values									
Endoscopic	7.0	16.0	5.0	5.5	4.0	0.129	0.887	0.786	0.698	**0.087**	**0.057**	0.031*	0.908	0.693	0.638
Diagnosis surgical triage	(1.5–2.5)	(4.0–8.0)	(1.75–2.75)	(1.25–0.0)	(1.–10.0)										
Endoscopic	18.0	21.0	19.0	12.0	12.0	0.334	0.506	0.987	0.709	0.667	0.474	0.399	0.591	0.526	0.771
Diagnosis surgery	(5.0–1.25)	(6.0–1.75)	(4.0–0.0)	(5.0–7.0)	(7.0–1.0)										

Data is shown as absolute numbers or as median with interquartile range (IQR) in brackets. Bold print indicates a trend. Lockdown (LD) in germany: 1. LD = 1. lockdown (22/03/20-04/05/20); 2. LD = 2. lockdown (02/11/20-26/12/20); 3. LD = 3. lockdown (27/12/20-09/05/21); no LD Covid = COVID-19 pandemic outside lockdown times; pre-covid = period before the covid pandemic in Germany (01/03/2018- 29/02/2020)

* *p*-values < 0.05 were considered significant

## Data Availability

The dataset of the study is available from the corresponding author on reasoned request.

## Code availability

Not used in the study.

## References

[1] World Health Organization (WHO) Novel coronavirus (2019-Ncov) advice for the public. Available online: https://www.who.int/emergencies/diseases/novel-coronavirus-2019/advice-for-public. Accessed on 1 October 2020

[2] Balkhair AA (2020) COVID-19 pandemic: a new chapter in the history of infectious diseases. Oman Med J 35(2):e123. [cited 2023 Mar 31]. Available from: https://www.ncbi.nlm.nih.gov/pmc/articles/PMC7171815/10.5001/omj.2020.41 PMC7171815 32328297

[3] Morris EJA, Goldacre R, Spata E, Mafham M, Finan PJ, Shelton J, Richards M, Spencer K, Emberson J, Hollings S, Curnow P, Gair D, Sebag-Montefiore D, Cunningham C, Rutter MD, Nicholson BD, Rashbass J, Landray M, Collins R, Casadei B, Baigent C (2021). Impact of the COVID-19 pandemic on the detection and management of colorectal cancer in England: a population-based study. Lancet Gastroenterol Hepatol.

[4] Hunger R, König V, Stillger R, Mantke R (2022) Impact of the COVID-19 pandemic on delays in surgical procedures in Germany: a multi-center analysis of an administrative registry of 176,783 patients. Patient Saf Surg 16:22. [cited 2023 Mar 31]. Available from: https://www.ncbi.nlm.nih.gov/pmc/articles/PMC9238103/10.1186/s13037-022-00331-y PMC9238103 35765000

[5] Muschol J, Strauss C, Gissel C (2022) COVID-19 related decline in cancer screenings most pronounced for elderly patients and women in Germany: a claims data analysis. J Cancer Res Clin Oncol 1–23. [cited 2023 Mar 31]. Available from: https://www.ncbi.nlm.nih.gov/pmc/articles/PMC9702775/10.1007/s00432-022-04433-z PMC9702775 36436091

[6] https://www.bundesgesundheitsministerium.de/fileadmin/Dateien/3_Downloads/K/Krebs/Krebsgeschehen_RKI.pdf

[7] Halfter K, Bauerfeind L, Schlesinger-Raab A, Schmidt M, Schubert-Fritschle G, Hölzel D, Engel J (2021). Colonoscopy and polypectomy: beside age, size of polyps main factor for long-term risk of colorectal cancer in a screening population. J Cancer Res Clin Oncol.

[8] Zauber AG, Winawer SJ, O’Brien MJ, Mills GM, Allen JI, Feld AD, Jordan PA, Fleisher M, Orlow I, Meester RGS, Lansdorp-Vogelaar I, Rutter CM, Knudsen AB, Mandelson M, Shaukat A, Mendelsohn RB, Hahn AI, Lobaugh SM, Palmer BS, Serrano V, Kumar JR, Fischer SE, Chen JC, Bayuga-Miller S, Kuk D, O’Connell K, Church TR (2023) Randomized trial of facilitated adherence to screening-colonoscopy versus sequential fecal-based blood test. Gastroenterology S0016–5085(23)00505-X 10.1053/j.gastro.2023.03.206 PMC10330012 36948424

[9] Zauber AG, Winawer SJ, O’Brien MJ, Lansdorp-Vogelaar I, van Ballegooijen M, Hankey BF, Shi W, Bond JH, Schapiro M, Panish JF, Stewart ET, Waye JD (2012) Colonoscopic polypectomy and long-term prevention of colorectal-cancer deaths. N Engl J Med 366(8):687–696 10.1056/NEJMoa1100370 PMC3322371 22356322

[10] Patel S, Issaka RB, Chen E, Somsouk M (2021) Colorectal cancer screening and COVID-19. Am J Gastroenterol 116(2):433–434 10.14309/ajg.0000000000000970 PMC7553028 33038127

[11] Gorin SNS, Jimbo M, Heizelman R, Harmes KM, Harper DM (2021). The future of cancer screening after COVID-19 may be at home. Cancer.

[12] Brenner H, Cardoso R, Heisser T, Hoffmeister M, Holleczek B (2022). Indications of substantial delay of colorectal cancer diagnoses due to COVID-19 in Germany. Lancet Reg Health Eur.

[13] Santoro GA, Grossi U, Murad-Regadas S, Nunoo-Mensah JW, Mellgren A, Di Tanna GL, Gallo G, Tsang C, Wexner SD, DECOR-19 Collaborative Group (2021) DElayed COloRectal cancer care during COVID-19 pandemic (DECOR-19): global perspective from an international survey. Surgery 169(4):796–807 10.1016/j.surg.2020.11.008 PMC7670903 33353731

[14] Clavien PA, Barkun J, de Oliveira ML, Vauthey JN, Dindo D, Schulick RD, de Santibañes E, Pekolj J, Slankamenac K, Bassi C, Graf R, Vonlanthen R, Padbury R, Cameron JL, Makuuchi M (2009). The Clavien-Dindo classification of surgical complications: five-year experience. Ann Surg.

[15] Doyle DJ, Hendrix JM, Garmon EH (2023) American Society of Anesthesiologists Classification. In: StatPearls [Internet]. Treasure Island (FL): StatPearls Publishing. [cited 2023 Mar 28]. Available from: http://www.ncbi.nlm.nih.gov/books/NBK441940/28722969

[16] Weiser MR (2018) AJCC 8th Edition: Colorectal Cancer. Ann Surg Oncol 25(6):1454–5. [cited 2023 Mar 30]. Available from: 10.1245/s10434-018-6462-1 10.1245/s10434-018-6462-1 29616422

[17] Vogel JD, Felder SI, Bhama AR, Hawkins AT, Langenfeld SJ, Shaffer VO, Thorsen AJ, Weiser MR, Chang GJ, Lightner AL, Feingold DL, Paquette IM (2022) The American Society of Colon and Rectal Surgeons clinical practice guidelines for the management of colon cancer. Dis Colon Rectum 65(2):148. [cited 2023 Mar 30]. Available from: https://journals.lww.com/dcrjournal/Fulltext/2022/02000/The_American_Society_of_Colon_and_Rectal_Surgeons.7.aspx#JCL-P-19 10.1097/DCR.0000000000002323 34775402

[18] Galon J, Mlecnik B, Bindea G, Angell HK, Berger A, Lagorce C, Lugli A, Zlobec I, Hartmann A, Bifulco C, Nagtegaal ID, Palmqvist R, Masucci GV, Botti G, Tatangelo F, Delrio P, Maio M, Laghi L, Grizzi F, Asslaber M, D’Arrigo C, Vidal-Vanaclocha F, Zavadova E, Chouchane L, Ohashi PS, Hafezi-Bakhtiari S, Wouters BG, Roehrl M, Nguyen L, Kawakami Y, Hazama S, Okuno K, Ogino S, Gibbs P, Waring P, Sato N, Torigoe T, Itoh K, Patel PS, Shukla SN, Wang Y, Kopetz S, Sinicrope FA, Scripcariu V, Ascierto PA, Marincola FM, Fox BA, Pagès F (2014) Towards the introduction of the ‘Immunoscore’ in the classification of malignant tumours. J Pathol 232(2):199–209. [cited 2023 Mar 30]. Available from: https://www.ncbi.nlm.nih.gov/pmc/articles/PMC4255306/10.1002/path.4287 PMC4255306 24122236

[19] Diers J, Acar L, Wagner JC, Baum P, Hankir M, Flemming S, Kastner C, Germer CT, L’hoest H, Marschall U, Lock JF, Wiegering A (2022) Cancer diagnosis is one quarter lower than the expected cancer incidence in the first year of COVID-19 pandemic in Germany: a retrospective register-based cohort study. Cancer Commun (Lond) 42(7):673–6 10.1002/cac2.12314 PMC9257987 35633279

[20] Diers J, Acar L, Baum P, Flemming S, Kastner C, Germer CT, L’hoest H, Marschall U, Lock JF, Wiegering A (2021) Fewer operations for cancer in germany during the first wave of COVID-19 in 2020–a cohort study and time-series analysis. Dtsch Arztebl Int 118(27–28):481–2 10.3238/arztebl.m2021.0265 PMC8456444 34491160

[21] Uttinger KL, Diers J, Baum P, Hankir M, Germer CT, Wiegering A (2023). Impact of the COVID pandemic on major abdominal cancer resections in Germany: a retrospective population-based cohort study. Int J Surg.

[22] Voigtländer S, Hakimhashemi A, Inwald EC, Ortmann O, Gerken M, Klug SJ, Klinkhammer-Schalke M, Meyer M, Müller-Nordhorn J (2021). The impact of the COVID-19 pandemic on cancer incidence and treatment by cancer stage in Bavaria. Germany Dtsch Arztebl Int.

[23] Radulovic RS, Cuk VV, Juloski JT, Arbutina DD, Krdžic ID, Milic LV, Kenic MV, Karamarkovic AR (2021). Is colorectal cancer stage affected by COVID-19 pandemic?. Chirurgia (Bucur).

[24] Aguiar S, Riechelmann RP, de Mello CAL, da Silva JCF, Diogenes IDC, Andrade MS, de Miranda Marques TMD, Stevanato PR, Bezerra TS, Silva MLG, Lopes A, Curado MP (2021). Impact of COVID-19 on colorectal cancer presentation. Br J Surg.

[25] Cui J, Li Z, An Q, Xiao G (2022). Impact of the COVID-19 pandemic on elective surgery for colorectal cancer. J Gastrointest Cancer.

[26] Choi JY, Park IJ, Lee HG, Cho E, Kim YI, Kim CW, Yoon YS, Lim SB, Yu CS, Kim JC (2021). Impact of the COVID-19 pandemic on surgical treatment patterns for colorectal cancer in a tertiary medical facility in Korea. Cancers (Basel).

[27] Cuk P, Simonsen RM, Sherzai S, Buchbjerg T, Andersen PV, Salomon S, Pietersen PI, Möller S, Al-Najami I, Ellebaek MB (2023) Surgical efficacy and learning curves of laparoscopic complete mesocolic excision with intracorporeal anastomosis for right-sided colon cancer: a retrospective two-center cohort study. J Surg Oncol 10.1002/jso.27230 36933189

[28] Mocan L (2021). Laparoscopic surgery for the treatment of colon cancer: the new standard?. Eur Rev Med Pharmacol Sci.

[29] Marusch F, Gastinger I, Schneider C, Scheidbach H, Konradt J, Bruch HP, Köhler L, Bärlehner E, Köckerling F, Laparoscopic Colorectal Surgery Study Group (LCSSG) (2001) Experience as a factor influencing the indications for laparoscopic colorectal surgery and the results. Surg Endosc 15(2):116–20. [cited 2023 Apr 2]. Available from: 10.1007/s004640000340 10.1007/s004640000340 11285950

[30] https://www.rki.de/DE/Content/InfAZ/N/Neuartiges_Coronavirus/Fallzahlen.html

[31] https://www.berlin.de/lageso/gesundheit/infektionskrankheiten/corona/tabelle-indikatoren-gesamtuebersicht/

[32] Flemming S, Hankir MK, Kusan S, Krone M, Anger F, Germer CT, Wiegering A (2021). Safety of elective abdominal and vascular surgery during the COVID-19 pandemic: a retrospective single-center study. Eur J Med Res.

[33] NIHR Global Health Unit on Global Surgery, COVIDSurg Collaborative (2022) Elective surgery system strengthening: development, measurement, and validation of the surgical preparedness index across 1632 hospitals in 119 countries. Lancet 400(10363):1607–17 10.1016/S0140-6736(22)01846-3 PMC9621702 36328042

